# Simultaneous determination of two serum tumor markers in assessing malignant melanoma patients

**DOI:** 10.1186/1479-5876-13-S1-P9

**Published:** 2015-01-15

**Authors:** Angela Sandru, Silviu Voinea, Eugenia Panaitescu, Madalina Bolovan, Adina Stanciu, Sabin Cinca, Alexandru Blidaru

**Affiliations:** 1 Department of Surgical Oncology, “Carol Davila” University of Medicine and Pharmacy; “Alexandru Trestioreanu” Oncologic Institute, Bucharest, Romania; 2 Department of Medical Informatics and Biostatistics ,“Carol Davila” University of Medicine and Pharmacy, Bucharest, Romania; 3 Department of Carcinogenesis and Molecular Biology, “Alexandru Trestioreanu” Oncologic Institute, Bucharest, Romania

## Background

The incidence of cutaneous malignant melanoma (MM) continues to raise despite extensive prevention programs. If localized disease can be cured, the prognosis of metastatic melanoma is grim. Therefore, reliable methods to detect patients at risk for disease progression are sought. Field of tumor markers has captured attention because it allows the first steps toward personalized medicine. A positive tumor marker has a limited ability to correctly detect all sick people, so it was proposed to simultaneously measure several markers in order to improve their performance [1,2]. S100 and Melanoma Inhibitory Activity (MIA) are most frequently used for monitoring MM patients. Both proteins have a high specificity and their expression correlates with body tumor burden [3].

## Materials and methods

Between 2009 and 2013, we determined MIA and S100 serum concentration in 120 patients with non-metastatic MM and 50 healthy donors, in order to compare diagnostic and prognostic potential of these two biomarkers. Both proteins were measured by a high sensitivity ELISA method. For S100, a threshold of 100 ng/L was accepted, as recommended by the kit manufacturer. Using the ROC curve, we estimated a MIA cut-off level of 9.4 ng/mL [4]. Patients were divided into 4 groups according to markers concentrations: both markers positive, both negative and one positive/one negative. Median, disease free and overall survival (OS) were estimated for each group.

## Results

Survival varied depending on the number and type of markers exceeding the cut-off. Median survival decreased in this order: from S100/MIA negative group to MIA negative/S100 positive, MIA positive/S100 negative, S100/MIA positive group. It seems that a MIA value above the cut-off has a negative impact on OS greater than an increased S100 value. Two years OS was significantly higher in MIA/S100 negative group compared with MIA/S100 positive one (81%/51%; p=0.05). Furthermore, patients with a single positive marker had a higher OS than those with both markers increased.

## Conclusions

Simultaneous use of S100 and MIA increased sensitivity of identifying MM patients irrespective of clinical stage. Several tumor biomarkers determination affords selection of those produced in high volume that will be further used in patients follow-up. Measuring both MIA and S100 allows outlining of an intermediate prognosis group of patients, represented by those with a single positive marker, who have a lower risk of relapse and death than those with both positive markers.
